# Twenty-Four Hour Non-Invasive Ambulatory Blood Pressure and Heart Rate Monitoring in Parkinson’s Disease

**DOI:** 10.3389/fneur.2013.00049

**Published:** 2013-05-15

**Authors:** Eva Stuebner, Ekawat Vichayanrat, David A. Low, Christopher J. Mathias, Stefan Isenmann, Carl-Albrecht Haensch

**Affiliations:** ^1^Autonomic Laboratory, Department of Neurology and Clinical Neurophysiology, Faculty of Health, HELIOS-Klinikum Wuppertal, University of Witten/HerdeckeWuppertal, Germany; ^2^Autonomic and Neurovascular Medicine Unit, Division of Brain Sciences, Faculty of Medicine, Imperial College London at St Mary’s HospitalLondon, UK; ^3^Autonomic Unit, Queen Square/Division of Clinical Neurology, National Hospital for Neurology and Neurosurgery, Institute of Neurology, University College LondonLondon, UK

**Keywords:** Parkinson’s disease, 24 h ambulatory blood pressure monitoring, orthostatic hypotension, supine hypertension, non-dipping, autonomic protocol, non-invasive, circadian rhythm

## Abstract

Non-motor symptoms are now commonly recognized in Parkinson’s disease (PD) and can include dysautonomia. Impairment of cardiovascular autonomic function can occur at any stage of PD but is typically prevalent in advanced stages or related to (anti-Parkinsonian) drugs and can result in atypical blood pressure (BP) readings and related symptoms such as orthostatic hypotension (OH) and supine hypertension. OH is usually diagnosed with a head-up-tilt test (HUT) or an (active) standing test (also known as Schellong test) in the laboratory, but 24 h ambulatory blood pressure monitoring (ABPM) in a home setting may have several advantages, such as providing an overview of symptoms in daily life alongside pathophysiology as well as assessment of treatment interventions. This, however, is only possible if ABPM is administrated correctly and an autonomic protocol (including a diary) is followed which will be discussed in this review. A 24-h ABPM does not only allow the detection of OH, if it is present, but also the assessment of cardiovascular autonomic dysfunction during and after various daily stimuli, such as postprandial and alcohol dependent hypotension, as well as exercise and drug induced hypotension. Furthermore, information about the circadian rhythm of BP and heart rate (HR) can be obtained and establish whether or not a patient has a fall of BP at night (i.e., “dipper” vs. non-“dipper”). The information about nocturnal BP may also allow the investigation or detection of disorders such as sleep dysfunction, nocturnal movement disorders, and obstructive sleep apnea, which are common in PD. Additionally, a 24-h ABPM should be conducted to examine the effectiveness of OH therapy. This review will outline the methodology of 24 h ABPM in PD, summarize findings of such studies in PD, and briefly consider common daily stimuli that might affect 24 h ABPM.

## Introduction

The motor symptoms of Parkinson’s disease (PD) are widely known and well studied. In the past 20 years non-motor symptoms, which include dysfunction of the autonomic nervous system have been increasingly defined and investigated. Non-motor symptoms are important to the patients and have been found to have a severe effect on quality of life (Antonini et al., 2012). A feature of an impaired autonomic nervous system are cardiovascular symptoms, such as orthostatic hypotension (OH) and supine hypertension, which are common (de Visser et al., 2002). Cardiovascular symptoms are, besides infections, the most frequent reason for PD patients being admitted to hospital (Willis et al., 2012). Autonomic dysfunction has previously been reported in the advance stage of PD but now it has become clear that autonomic dysfunction can be found even in the early stage of PD without any medication in a population based cohort (Muller et al., 2011). Several studies have reported that autonomic features may occur before the hallmark motor symptoms of PD (Abbott et al., 2001; Haensch et al., 2009; Hawkes et al., 2010; Asahina et al., 2012). Therefore, a correct diagnosis of cardiovascular autonomic function symptoms is crucial in order to manage them appropriately and possibly even detect PD at a pre-motor or an earlier motor stage. Several approaches exist to diagnose cardiovascular autonomic dysfunction, including laboratory and community-based methods. The aim of this review is to discuss community-based methods, with a particular focus on 24 h ambulatory blood pressure (BP) monitoring in the home setting and consider its advantages and limitations.

### Parkinson’s disease and cardiovascular autonomic function

The autonomic nervous system controls a range of processes vital for health and wellbeing. It is divided into the parasympathetic and sympathetic nervous systems which innervate nearly every organ in the human body (Asahina et al., 2012). Beat-to-beat control of BP as well as the perfusion of various organs are a vitally important task of the autonomic nervous system (Iodice et al., 2010). Physiologic feedback mechanisms via the baroreflex loops work through cranio-sacral parasympathetic and thoraco-lumbar sympathetic neural pathways to regulate BP and keep it at an appropriate level, depending on posture and various situations (Low and Mathias, 2011). A change in posture always requires an appropriate autonomic response as due to gravitational stress there is a shift of 500–700 ml of blood from central compartments to the legs, causing marked pressure differentials (Hainsworth, 1985; Mathias, 2002).

A variety of non-motor symptoms in PD are linked to the impairment of the autonomic nervous system. Symptoms due to an impaired autonomic nervous system can be caused by aging processes in addition to the Parkinsonism itself. The Braak staging hypothesis implicates that in PD, Lewy bodies invade autonomic centers and the dorsal motor nucleus of the glossopharyngeal and vagal nerves, the gastrointestinal submucosal plexus and the post-ganglionic sympathetic nervous system (Asahina et al., 2012). With increasing age, the sensitivity of the baroreceptors decreases which can cause a loss of BP control (Haensch and Jörg, 2005). Amongst others, this is a reason for why cardiovascular disorders increase with age and often affect PD patients since the majority of them are older than 50 years (Iodice et al., 2010).

Parkinson’s disease patients often suffer from more than one symptom caused by dysfunction of the autonomic nervous system (Goldstein, 2003). These symptoms can range from gastrointestinal, urinary and sexual problems, abnormal sweating, heat intolerance, and pupillary abnormalities to cardiovascular symptoms (Goldstein, 2003; Asahina et al., 2012). Cardiovascular symptoms can manifest themselves in features such as supine hypertension and various forms of hypotension, such as orthostatic, postprandial, and exercise-induced hypotension. Hypotension, in its various forms, in PD, can be due to a variety of mechanisms, including, neurodegenerative processes in the dorsal vagus nerve (Jost, 2012), decreased post-ganglionic sympathetic innervation and central lesions in the upper brainstem which affect baroreflex function (Low et al., 2012).

### Orthostatic hypotension in Parkinson’s disease

Orthostatic hypotension is defined as a decrease of systolic BP by ≥20 mmHg or diastolic BP by ≥10 mmHg in 3 min after standing upright or at a minimum of 60° head-up-tilt (HUT) (Lahrmann et al., 2006; Asahina et al., 2012). In the general population of the elderly (aged 65 and above) the prevalence of OH has been estimated to be within a range of 5–36.3% (Alli et al., 1992; Low, 2008), and even up to 50% (Lipsitz, 1983). The wide variety in prevalence could be caused by diverse factors, such as different protocols or level of orthostatic stress. But even when the criteria for OH are met, patients do not necessarily suffer from symptoms, because the fall in BP can be tolerated. A reason for this might be habituation to slowly decreasing levels of BP due to an improvement of cerebral autoregulation which is known to occur in patients with autonomic failure (Brooks et al., 1989). This may explain why many PD patients with OH are asymptomatic (Iodice et al., 2010).

In patients with OH the ability to increase vascular resistance when standing is absent or impaired which leads to increased venous pooling in the lower limbs, reduced stroke volume, and decreased cardiac output (Smit et al., 1999). This may result in cerebral, cardiac, renal, muscular hypoperfusion, and cause attendant symptoms, as well as weakness, lethargy, fatigue, falls (Mathias and Kimber, 1999), and “coat hanger pain” in back and shoulders (Mathias et al., 1999). Furthermore, in elderly patients who suffer from certain neurological disorders, existing OH can worsen the prognosis of the disease and increase mortality (Low, 2008). Speed of positional change, time of day (worse in the morning), prolonged recumbence, warm environments (hot weather, central heating, hot bath) causing cutaneous vasodilatation, raising intrathoracic pressure (micturition, defecation, coughing), food or alcohol ingestion because of splanchnic vasodilatation, physical exertion maneuvers which lead to skeletal muscle vasodilatation and positions (bending forward, abdominal compression, leg crossing, squatting, activating calf muscle pump), drugs with vasoactive properties (including dopaminergic agents) (Iodice et al., 2010) are all such factors and can lead to possible low BP readings or OH (see Table 1).

**Table 1:** **Factors influencing postural (orthostatic) hypotension, adapted from Mathias et al. (2013)**.

**FACTORS INFLUENCING POSTURAL (ORTHOSTATIC) HYPOTENSION**	
Speed of positional change	
Time of day (worse in the morning)	
Prolonged recumbency	
Warm environment (hot weather, central heating, hot bath)	
Raising intrathoracic pressure – micturition, defaecation, or coughing	
Food and alcohol ingestion	
Physical exertion	
Maneuvers and positions (bending forward, abdominal compression, leg crossing, squatting, activating calf muscle pump)*	
Drugs with vasoactive properties (including dopaminergic agents)	
**NON-NEUROGENIC CAUSES OF POSTURAL HYPOTENSION**	
Low intravascular volume	
Blood/plasma loss	Hemorrhage, burns, hemodialysis
Fluid/electrolyte	Inadequate intake – anorexia nervosa
	Fluid loss – vomiting, diarrhea, losses from ileostomy
	Renal/endocrine – salt-losing nephropathy, adrenal insufficiency (Addison’s disease), diabetes insipidus, diuretics
Vasodilatation	Drugs – glyceryl trinitrate
	Alcohol
	Heat, pyrexia
	Hyperbradykinism
	Systemic mastocytosis
	Extensive varicose veins
	Cardiac impairment
Myocardial	Myocarditis
Impaired ventricular filling	Atrial myxoma, constrictive pericarditis
Impaired output	Aortic stenosis

*These maneuvers usually reduce the postural fall in blood pressure, unlike the others.

The majority of PD patients suffer from at least one cardiovascular symptom. This has been found when looking for entirely unremarkable results in cardiovascular autonomic function in PD patients, which were only found in 3 out of 100 patients when conducting a variety of cardiovascular autonomic function tests (AFT) (Jost, 1999). However, this finding depends on the definition of “cardiovascular symptoms” and should be compared with the prevalence of abnormal cardiovascular findings in healthy controls. Unlike the general population, where the incidence of hypertension increases with age, PD patients only rarely suffer from hypertension and tend to have lower BP readings (Jost, 2012). Findings about the prevalence of OH in PD vary widely in literature, but a recent meta-analysis of 25 studies calculated that about 30% of PD patients suffer from OH (Velseboer et al., 2011). There is also the notion that OH may occur before the hallmark motor symptoms (Goldstein et al., 2000). Compared to patients who do not show OH, those with OH were found to be of older age (Lipsitz, 1983; Allcock et al., 2004). Consistent with this finding, the incidence of OH rises with age, disease severity, and (dopaminergic) drug therapy in PD (Goldstein, 2003; Verbaan et al., 2007; Iodice et al., 2011). A study by Oh et al. (2012) found that OH does not correlate with motor symptom severity.

Parkinson’s disease patients with OH have impaired baroreflex-cardiovagal function (Goldstein, 2003) and generally have a loss of cardiac sympathetic innervation. However no correlation was found between Cardiac MIBG [(123)I-metaiodobenzylguanidine)] uptake and OH or other autonomic findings in PD (Haensch et al., 2009; Oka et al., 2011), suggesting that cardiac sympathetic innervation decreases independently (Haensch, 2011). Usually patients with OH have low norepinephrine levels which suggests that a BP fall could also be caused by an extra cardiac impairment (Goldstein et al., 2003a). Unlike in PD, multiple system atrophy (MSA) patients have intact cardiac sympathetic innervation (Goldstein, 2003). Both groups of patients (PD and MSA) tend to present with abnormal nocturnal BP regulation, which is also linked to OH (Iodice et al., 2010). MSA is a clinically progressive neurodegenerative disorder which is characterized by Parkinsonism, cerebellar ataxia, and autonomic dysfunction (Gilman et al., 2008). The symptoms of MSA often overlap with those of PD, which can make it difficult for doctors to be able to distinguish between the two conditions. OH is a cardinal and diagnostic feature of MSA and it is therefore recommended that PD patients showing signs of OH should always, as a precaution, be tested for MSA (Gilman et al., 2008), in order to discriminate between the two conditions.

Diagnosis and treatment of OH in PD are important, since OH could lead to falls and injuries and consequently increased morbidity (Jamnadas-Khoda et al., 2009). The usual approach to achieve a diagnosis of OH is a standing test (also known as Schellong test) or passive HUT in an autonomic laboratory. Acquiring a detailed medical history of a patient in order to exclude OH is essential but not sufficient by itself. Because typical symptoms connected to OH, such as light-headedness, dizziness, visual disturbances, impaired cognition, angina pectoris, etc. (Haensch et al., 2009) are not specific to OH and might be omitted or not be reported by some patients suffering from OH (Jamnadas-Khoda et al., 2009). Sometimes patients might only report increased fatigue which could also be a sign of OH (Streeten and Anderson, 1998). Independently of PD, elderly people often tend to suffer from dizziness and orthostatic symptoms which may be due to polyneuropathy that leads to a loss of peripheral nerve fibers (including autonomic nerve fibers) (Haensch and Jörg, 2005). Therefore, reported dizziness does not necessarily indicate OH (caused by PD) and should be further clarified. It is important to always look for other possible causes of OH as well as (autonomic) polyneuropathy, which could possibly be found coincidently in PD due to advanced age. But even though PD patients often suffer from OH symptoms, such as dizziness and falls, they only very rarely actually lose consciousness (Jost, 2012).

A standing test, in which BP is measured every minute and a patient is simply asked to lie still for 10 min and afterward stand in an upright position for 10 min might be a good alternative to a HUT for which extensive equipment such as an electronically driven tilt-table and software are needed. Some even argue that the standing test is more adequate for measuring OH in PD patients since it allows for appropriate physiological responses (Jost, 2012). The standing test might not always be sufficient because even though it is very specific it has a low sensitivity compared to the HUT test (Winker et al., 2005). Therefore the guideline developed by the European Federation of Neurological Sciences (EFNS) recommends the HUT if the standing test is negative and OH is suspected (Lahrmann et al., 2006). It might be useful to generally tilt patients with motor impairments such as PD in order to exclude OH. The HUT can be extended to an AFT which also measures sympathetic vasoconstrictor and parasympathetic responsiveness using various pressor tests (including isometric exercise, cold pressor, and mental arithmetic tests), Valsalva maneuver, hyperventilation, deep breathing with ECG, and beat-to-beat BP measurement in order to further establish whether cardiovascular autonomic function is normal or abnormal (Mathias et al., 2013). OH can occur after the standard 3 min criteria and this is called late OH (L-OH) and occurs in about 20% of PD patients (Iodice et al., 2010). Therefore, some laboratories tilt PD patients over a longer period of time, such as 20 min, and conduct an additional standing test in order to compare the results of the two tests and possibly reveal a masked OH. A third, but not yet very prevalent option to diagnose OH as well as l-OH is 24 h ambulatory blood pressure monitoring (ABPM) in the home setting, which allows an investigation of several aspects in daily life and possible factors that may influence BP, outside of the clinical laboratory over a prolonged period of time.

## Twenty-Four Hour Ambulatory Blood Pressure Monitoring

### Circadian rhythms

Circadian rhythms in humans are controlled by the suprachiasmatic nuclei (SCN) in the hypothalamus (Buijs et al., 2003; Dibner et al., 2010; Golombek and Rosenstein, 2010). This endogenous clock and the secondary clocks in nearly every body cell (Dibner et al., 2010) are set to an approximate 24 h cycle (Golombek and Rosenstein, 2010). To maintain this 24 h rhythm and stay synchronized with the environment, it needs input from the outside. This is achieved by photic cues received by the retina and signaled to the SCN. These photic cues receive input from the light-dark cycle, a so called Zeitgeber (“time giver”) signal (Golombek and Rosenstein, 2010), which resets the SCN clock (Liu et al., 1997) and is one way to allow the clock to co-ordinate with the surroundings (Dibner et al., 2010). The SCN is consequently interconnected with the paraventricular nucleus (PVN) which is situated in the hypothalamus and plays a crucial role in coordinating autonomic function, such as stress, metabolism, and in this context most importantly, cardiovascular autonomic function (Ferguson et al., 2008). This way, the SCN proactively influences circadian physiology (Dibner et al., 2010) anticipating daytime and preparing the body for the upcoming day by raising glucose and cortisol levels as well as heart rate (HR) (Buijs et al., 2003). Conversely, melatonin, a hormone that causes drowsiness, is secreted by the pineal gland and is likewise controlled by the SCN via the PVN (Isobe and Nishino, 2004). Melatonin levels decrease in the morning (Buijs et al., 2003), depending on the light-dark cycle (Altun and Ugur-Altun, 2007).

**Figure 1:**
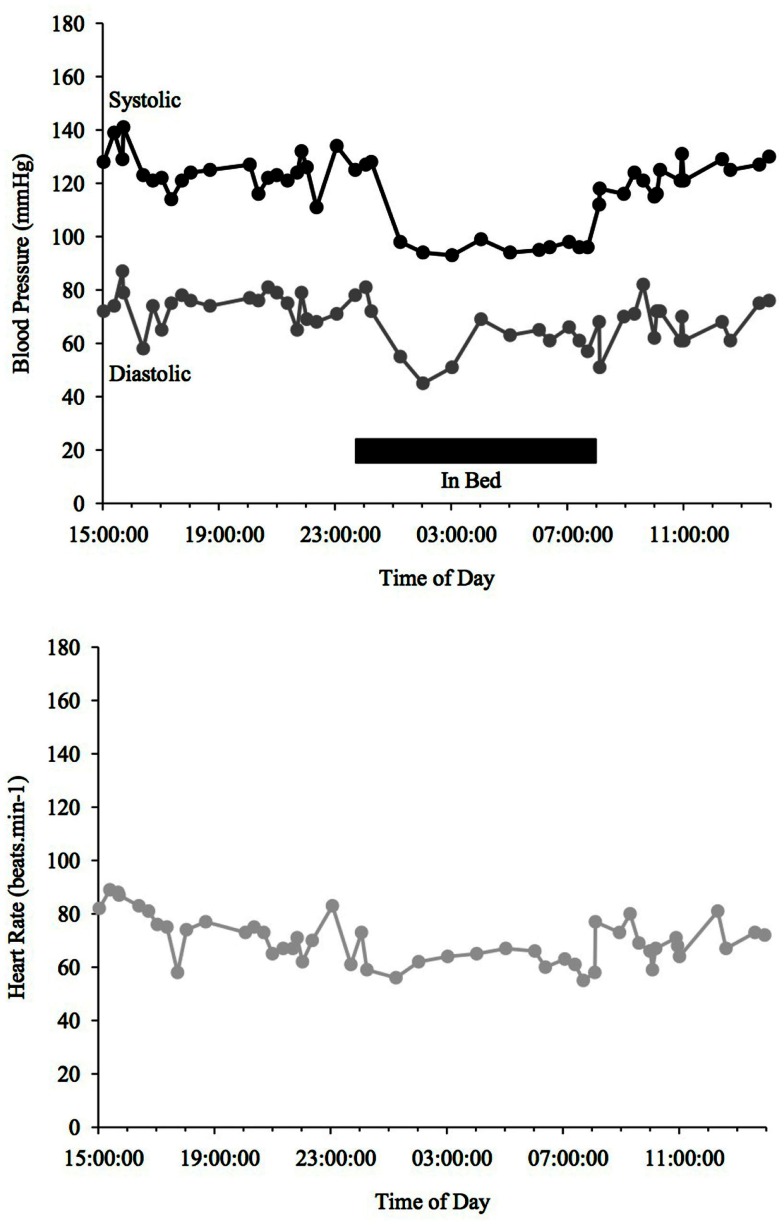
**Blood Pressure (upper line systolic, lower line diastolic) and heart rate profiles during 24 h ABPM of a healthy individual; dipper, showing more than a 10% drop in BP at night time**. Black bar indicates sleep.

Blood pressure and HR changes during a 24 h period depend on the sleep-activity rhythm and on endogenous circadian rhythms that affect vascular and cardiac functions (Fabbian et al., 2012). MAP (mean arterial pressure) BP typically declines ∼10–20% when going from an awake state to being asleep (Snyder et al., 1964) (see Figure 1). People with such a nocturnal fall of BP are called “dippers” as opposed to those, whose BP does not fall adequately (see Figure 3) or is even reversed (see Figure 4) who are called “non-dippers.” Furthermore, the circadian BP rhythm can exhibit two daytime peaks, one in the morning after getting up (∼08:00–10:00 h) and one in the evening, roughly 10–12 h later than the first one (Hermida et al., 2001). Another well recognized marker of circadian rhythms is the changes in body core temperature, which is also affected by the sleep-wake cycle and displays, similar to BP and HR, a dip at night and peak in the evening (Waterhouse et al., 2005).

### PD and circadian rhythms

**Figure 2:**
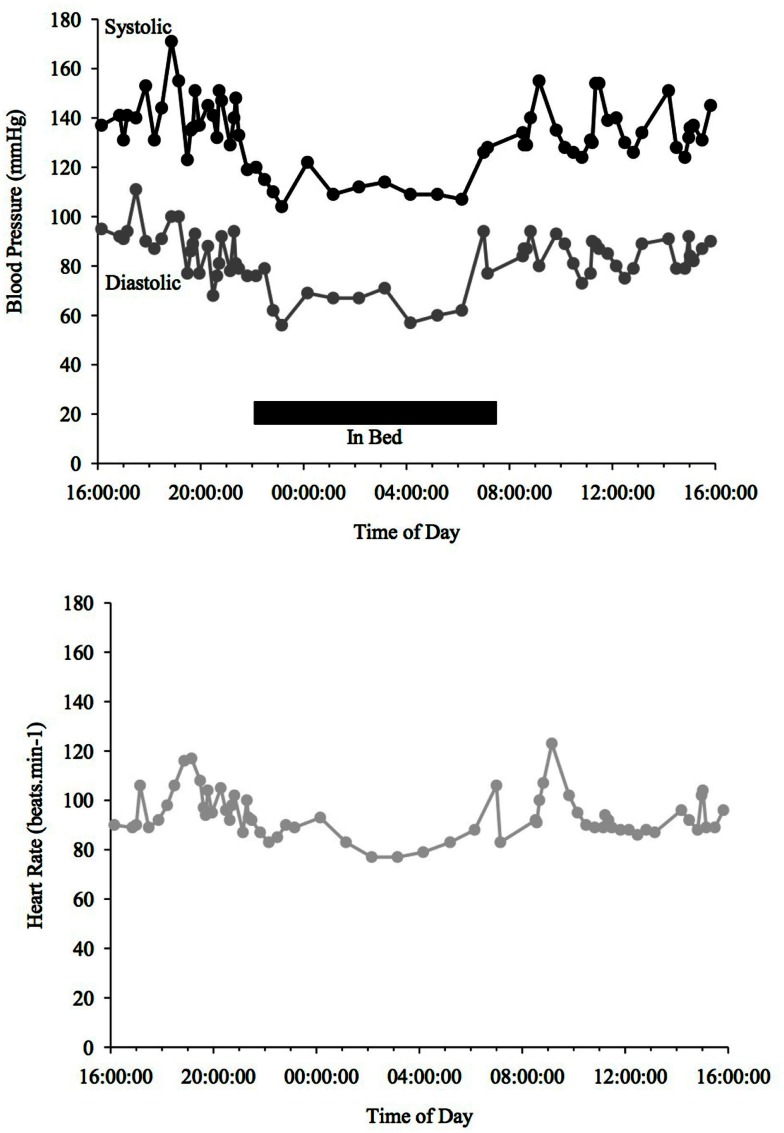
**Blood Pressure (upper line systolic, lower line diastolic) and heart rate profiles during 24 h ABPM of a PD patient; dipper, showing more than a 10% drop in BP at night time**. Black bar indicates sleep. Peaks in BP relate to agitation or increased tremor.

Circadian rhythms are susceptible to a variety of influencing factors, since they are so delicately organized. It is therefore comprehensible that they can also be affected by PD (see Figure 2). While no direct link between PD and decreased melatonin has been found, it is known that melatonin levels recede with age (Altun and Ugur-Altun, 2007), which should therefore also apply to PD patients. Administering 50 mg of melatonin improved total night-time sleep in PD patients when compared to a PD placebo control group (Dowling et al., 2005).

**Figure 3:**
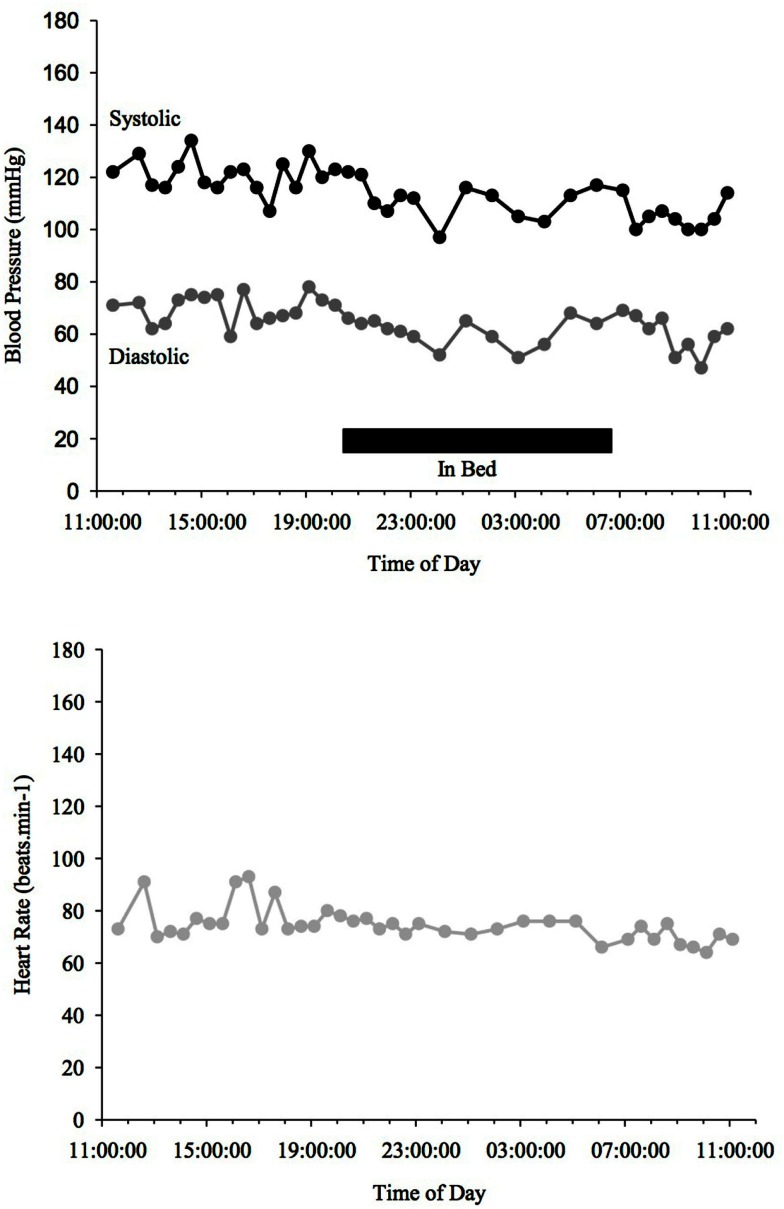
**Blood Pressure (upper line systolic, lower line diastolic) and heart rate profiles during 24 h ABPM of a PD patient, non-dipper, not showing a drop (<10%) of BP at night time**. Black bar indicates sleep.

**Figure 4:**
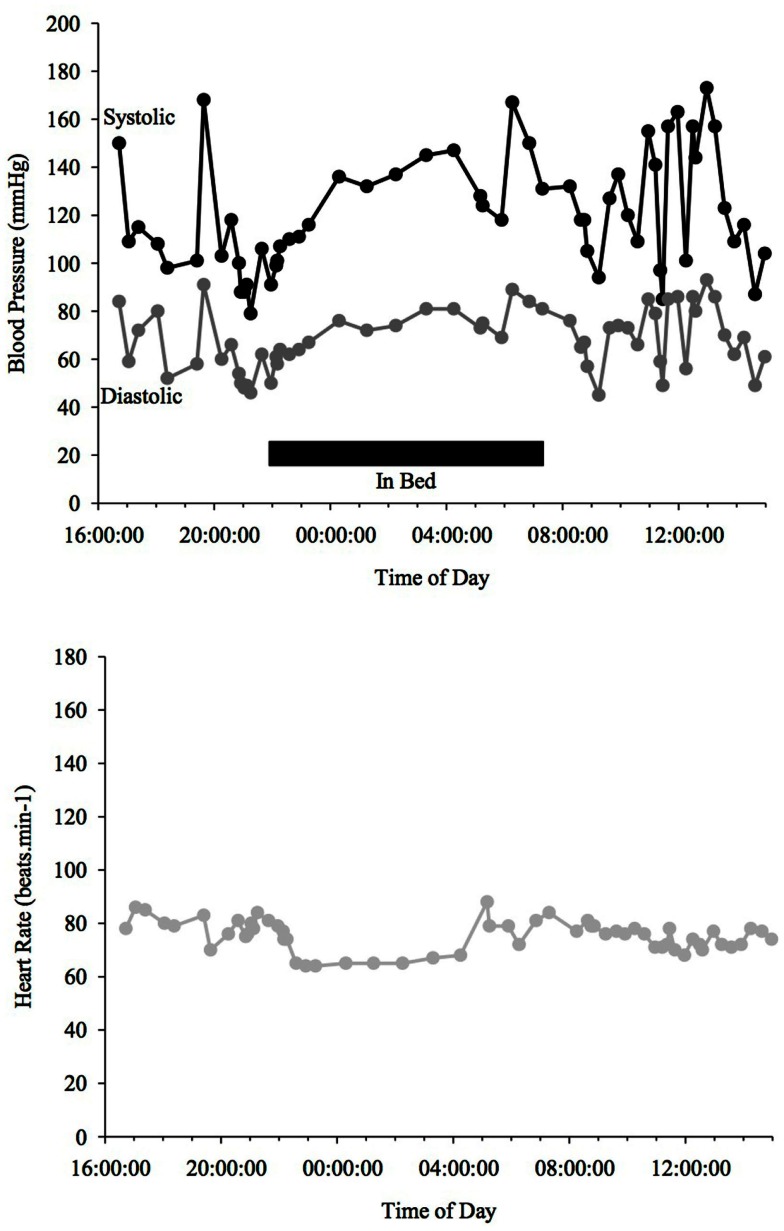
**Blood Pressure (upper line systolic, lower line diastolic) and heart rate profiles during 24 h ABPM of a PD patient with a reversed BP profile**. BP increases at night time in contrast to a dipper. Black bar indicates sleep.

Although the feature of acute OH in PD has been well researched, there are very few studies of the BP/HR circadian rhythms in PD. Motor activity levels, when measured with an actigraph (a non-invasive method of monitoring human rest/activity cycles) are reversed in PD patients, which means they are higher at night-time when in bed than during the day (Niwa et al., 2011). This is concordant with sleep disorders and daytime sleepiness that are often present in PD (see below) and may also not create an ideal foundation for appropriate BP and HR 24 h rhythms. A study looking at 7 PD and 14 probable MSA patients, found that core temperature does fall at night in patients with PD, contrary to MSA patients whose core temperature does not decrease nocturnally (Pierangeli et al., 2001).

### Twenty-four hour ABPM

Ambulatory BP monitoring using a standard upper arm cuff method over 24 h may be an extremely useful tool in screening cardiovascular autonomic function, especially BP variability and OH. Some may even consider it superior to HUT (Ejaz et al., 2006). The advantage of ABPM compared to a 3 min HUT is firstly the longer time frame it is conducted in, therefore allowing to screen BP not only at one specific point of a day. It furthermore rules out the effects of “white coat hypertension” and confounding variables of artificial clinical surroundings, to which elderly patients are more prone (Gerstner et al., 2006; Pickering et al., 2008). In a clinical setting, patients may not have OH because the fall in BP may occur later (L-OH) or only after daily stimuli such as food ingestion. A 24-h BP recording may therefore be a more economic and simple way to test for OH compared to routinely tilting patients, which requires a laboratory and a clinical autonomic scientist or nurse specialist. ABPM additionally grants insight into a patient’s daily routine, which allows for investigation of other factors influencing OH, such as differences in time of day, temperature variations, raising of intrathoracic pressure (such as coughing or defecation), exercise, reaction to medication, BP changes after food or alcohol ingestion (Iodice et al., 2010). Twenty-four hour ABPM also allows monitoring of a patient’s circadian rhythm, which reveals important information about BP characteristics at night, which can indicate if the patient is a dipper vs. a non-dipper. It has also been reported that ABPM better predicts the risk of morbid events than BP taken in a clinical setting (Pickering et al., 2005).

Other options to measure BP over a period of 24 h do exist, but there is no evidence for reasonable use of them in a community-based setting. One such alternative option is 24 h invasive BP measurements such as a brachial artery catheter (Imholz et al., 1993; Omboni et al., 1998). This is naturally the most precise way of measuring changes in BP, but can only be used in a hospital setting, since it needs to be monitored by health care professionals. A less invasive alternative would be 24 h recording using digital photoplethysmography using the Penaz method, which continuously measures beat-to-beat finger BP. This has been found to generate minimal error compared to brachial artery catheter BP measurements (Omboni et al., 1998). However, to date there is no research published on its use in a home setting and the equipment is often cumbersome and very technical. Therefore the well-established 24 h ABPM is recommended since it is easier to use, especially for older patients. A 24-h ABPM may not only be helpful to find causes of OH, but it is also of high importance for controlling OH therapy and assessing the effects of treatment (Iodice et al., 2010). Because OH may result from medication it is recommended to conduct an ABPM before and after starting a new therapy (Lahrmann et al., 2006).

It might be argued though that 24 h ABPM is not sufficient by itself to diagnose OH, which should always be secured by a HUT or AFT. ABPM only gives a good overview and might facilitate findings of potential other causes of OH, such as postprandial OH, but it only directs the doctor in the right direction and findings should always be confirmed with a HUT, meal-test, exercise test, etc depending on the circumstances. Another important aspect is that 24 h ABPM is more widely accessible than a HUT does not necessarily require referral to a specialist center and can therefore be conducted by a general practitioner. A combination of a 24-h ABPM in the home setting potentially followed by a HUT or an AFT in case of abnormal findings could provide the most thorough approach (see Chart 2).

### ABPM methodology

A well-structured ABPM can provide a range of diverse data about a patient, but when it is not done correctly, wrong conclusions may be drawn. A variety of factors should be considered when administering ABPM with PD patients, which will be discussed below.

The American Heart Association (AHA) recommends single BP measurements to be taken every 15–30 min during a 24-h period (Pickering et al., 2005). However, most published studies report measurements taken every 15–30 min during daytime and every 20–60 min at night (Urbina et al., 2008). Even though taking more regular measurements provides more data, changing the frequency of measurements at night time should be considered, since having BP taken at night might disrupt sleep and produce confounding variables and spurious results when assessing dipper vs. non-dipper status. But, having enough readings is crucial in order to effectively evaluate test results. The AHA proposes 50–100 readings per 24 h period (Pickering et al., 2005) whilst the British Hypertension Society considers as little as 14 valid measurements during day time and 7 at night time sufficient (O’Brien et al., 2000). Unsuccessful readings will have to be expected; a rate of 85% of successful readings is considered suitable for analysis (Head et al., 2012). A failed reading may be due to movement during measurement, incorrect cuff placement, weak or irregular pulse, or several other reasons (Head et al., 2012). The potential effects of movement in producing failed readings is of particular concern in PD patients due to tremor. Good compliance is therefore crucial and it is necessary to explain why it is important to keep the arm as still as possible when a measurement is taken.

Ideally an ABPM is conducted at home in a routine daily environment instead of a hospital setting. This is due to the fact that patients spend most of their time in a sitting or lying down position when hospitalized, which may not be normal for them. Even sleeping patterns might be influenced because of increased daytime rest and nocturnal distractions in a hospital ward setting. Therefore, measuring BP at home and instructing the patient to live a regular day as far as possible can provide more reliable data. To gain maximum insight into the BP readings, it is important to provide the patient with an autonomic protocol (including a diary) in order for them (or a relative/carrier) to record their activities and symptoms and therefore be able to make a connection between activities and BP changes during episodes. The format of such diaries used varies considerably. Such diaries should not only enquire about bed time/time when the patient fell asleep (including questions about the quality of sleep), time of awakening and time of medication taken (and what kind of medication) but also provide opportunities for the patient to record BP when symptomatic and also during various daily activities, such as sitting and standing throughout the day (i.e., postural change), before and after food ingestion, and exercise, as well as during and after medication (Mathias et al., 2013). An autonomic protocol/diary allows for a better screening of OH and other related features of cardiovascular autonomic dysfunction, e.g., postprandial hypotension (PPH).

It is essential to record at what time the patient went to bed and when they woke up, as well as quality of sleep in order to be able to evaluate the circadian BP pattern and detect if a patient is a dipper vs. a non-dipper. Another option is to equip patients with an accelerometer which measures physical activity and in some, even the position of the patient (Charbonnier et al., 1999). In addition, it can give more objective information then a self-kept activity diary, which is subjective to the patient. A variety of such devices have been developed, but they are not yet as widely and affordably accessible as current ABPMs.

A variety factors can worsen and influence OH, many of which can be detected during a 24 h BP measurement if an autonomic protocol is kept. Others may only be detected by asking the patient direct questions and obtaining a good history of past symptoms (please refer to Table 1).

## Twenty-Four Hour ABPM in PD

Many 24 h ABPM studies have been performed in PD, but mostly in a clinical setting, and only rarely in a home setting (see Table 2). Only a few studies (e.g., Ejaz et al., 2006; Schmidt et al., 2009) used a diary/autonomic protocol which was given to patients in order to be able to match BP readings to positions or activities. To date, no study has had patients perform a standing test or exercise test during the 24 h ABPM or to ascertain the occurrence of OH in a home setting.

**Table 2:** **An overview of key findings 24 hr ABPM studies in PD patients**.

Study title (reference)	Type of PD	Sample size	Setting	Technique	Treatment	% Of non-dipping	Findings
Brevetti et al. (1990)	PD	5 PD, 5 control	Clinical	Ambulatory intra-arterial BP measurement	Discontinued 15 days in advance	–	Mean 24 h BP was lower in PD patients than controls
Senard et al. (1992)	PD (± OH)	19 with OH, 19 without OH	Clinical	ABPM	l-DOPA, benserazide; bromocriptine	94.7% (PD + OH), 31.6% (PD − OH)	Average BP at night was higher and BP variability was higher during day than night in patients with OH (*p* < 0.05)
Muhl et al. (1997)	PD (± psychosis)	32	Clinical	ABPM, 15 min interval	l-DOPA	–	Psychosis in PD is correlated with low BP at night
Hakamaki et al. (1998)	PD (± OH)	20 PD, 21 controls	–		Fludrocortisone	40% (eight Patients on fludrocortisone were non-dipper)	PD patients without fludrocortisone had lower BP readings during day and night than PD on fludro
Plaschke et al. (1998)	PD (± OH)	13 PD, 11 PD + OH	Clinical	ABPM, 20 min interval during day, 30 min at night-time	All treatment discontinued at least 3 days prior to testing	–	Night-time BP was higher in 82% of PD + OH patients, higher supine BP in PD + OH than PD
Ejaz et al. (2006)	PD	13	Clinical	ABPM + Diary	Levodopa/carbidopa Selegiline Gabapentin, R-Blocker, calcium-channel-blocker	92.3%	postprandial hypotension, nocturnal hypertension in 100% of subjects
Schmidt et al. (2009)	PD	23 PD, 26 controls (25 PSP, 25 MSA)	Home setting	ABPM + Diary, 15 min interval during day, 30 min at night	Dopamine, dopamine antagonis, pramipexole, pergolid, Cabergoline, ropinirol e,a-dihydroergocryptine, MAO inhibitor, COMT inhibitor. Amantadine	48% (22% reversed)	Nocturnal BP regulation was pathological in PD 48%, PSP 40%, MSA 68% vs. Control 8%
Sommer et al. (2011)	PD	40	Home setting	ABPM	Antihypertensives	88%	No correlation between non-dipping and anti-hypertensive treatment; 95% of PD + OH patients were non-dipper and 79% of PD without OH
Oh et al. (2012)	Early PD (± OH)	52 PD, 17 PD + OH	–	ABPM	Antihypertensives	79.71%	The percentage of patients with hypertension was higher in the non-dipper group

Most studies investigating 24 h ABPM in PD focus on BP only, but HR may be of importance as well since it might provide a good overview of cardiovascular parameters (Mastrocola et al., 1999), since it has been found to decrease or increase accordingly with BP readings at night (Schmidt et al., 2009). In 24 h ABPM studies in PD patients it has been found that the average BP throughout 24 h is typically lower in patients with PD than controls (Brevetti et al., 1990) and those patients with OH have higher BP readings at night than those without OH (Senard et al., 1992; Plaschke et al., 1998) and also have a lower HR variability (Senard et al., 1992). Nocturnal BP regulation in PD patients was often found to be pathological because it does not drop or even increases at night in a 24 h ABPM study (Schmidt et al., 2009). This is consistent with the findings of another study which showed that all PD patients tested had PPH and nocturnal hypertension (Ejaz et al., 2006). In a 24 h ABPM study PD patients with hypertension were found to be more prone to non-dipping than normotensive participants (Oh et al., 2012). Even anti-hypertensive medication might not have an effect on the 24 h BP profile of PD patients, since a different study found no correlation between anti-hypertensive treatment and non-dipping, e.g., those on hypertensive medication were not more prone to non-dipping at night (Sommer et al., 2011). Another study found that PD patients who were treated with fludrocortisone have higher BP readings throughout 24 h than those who were not on fludrocortisone (Hakamaki et al., 1998).

It has been reported that PD patients who have low BP readings at night and who are dippers (mean ± SD SBP/DBP126 ± 18/74 ± 10 mmHg) are more prone to psychosis than those who have high nocturnal BP (Muhl et al., 1997).

All of the studies described above looking at 24 h BP measurements in PD detected OH in their participants, not necessarily through the 24 h monitoring itself but through a HUT.

### Postprandial hypotension

Postprandial hypotension in PD is known to worsen Parkinsonian symptoms such as tremor, bradykinesia, and gait difficulties, which can be extremely debilitating for patients (Chaudhuri et al., 1997). During 24 h ABPM, PPH, a symptom often found in PD (Mehagnoul-Schipper et al., 2001), might become evident. Furthermore, PPH or even postprandial with no fall in BP might unmask or exacerbate OH in PD patients which can be assessed during 24 h monitoring by performing a stand test for a period of 10 min, after a meal and noting any symptoms in the diary. However, a 24-h BP measurement can only provide preliminary information on whether PPH may be present; it is not a strong enough measure to make a diagnosis. Therefore, it should always be verified with a formal meal challenge in a laboratory setting (Iodice et al., 2010).

Nozaki et al. (1993) found a prevalence of PPH in PD of 61% in 14 patients. Another, more recent, study investigated PD patients during a standardized meal and standing test and found that PPH may even be more common than OH in (elderly) PD patients, compared to healthy controls (82% PPH in PD, 41% PPH in healthy controls, compared to 13% OH in PD, 6% in healthy controls) (Mehagnoul-Schipper et al., 2001). The same study showed that Levodopa did not have an effect on PPH, only disease severity did (Mehagnoul-Schipper et al., 2001). However, these studies were all conducted by testing patients in a clinical setting without using 24 h ABPM recording. Ejaz et al. (2006) however found a PPH prevalence of 100% in 13 PD patients using a 24 h ABPM with an autonomic protocol in a clinical setting. Alcohol may also exacerbate postural hypotension (Narkiewicz et al., 2000). A recent study found that alcohol consumption leads to a decline in the vasoconstrictor response to orthostatic stimuli (Narkiewicz et al., 2000). It is therefore crucial not only to examine the BP responses to food ingestion, but also alcohol intake (Mathias and Kimber, 1999).

A first step to reduce symptoms of PPH might be to ingest smaller and more frequent meals and less carbohydrates throughout the day in order to reduce the impact of a big meal while still eating enough to maintain a healthy weight. As a pharmacological treatment, octreotide could be considered (Albillos et al., 1994).

### Responses to exercise in PD

Alterations of cardiovascular responses during exercise can be evident in PD patients, especially if autonomic failure is present, but research is limited. A recent review (Low et al., 2012) summarized three papers that reported that PD patients showed reduced elevations in BP, HR, and noradrenaline when exercising (cycling) compared to age-matched healthy controls (Reuter et al., 1999; Werner et al., 2006; DiFrancisco-Donoghue et al., 2009). These findings suggest PD patients show reduced hemodynamic responses when exercising. Another study found that BP reactivity in healthy individuals to exercise is highest in the morning, but the reason for this is not dependent on sleep (Jones et al., 2008). Participants who slept in the afternoon before being tested showed the same BP reactivity as those participants who did not sleep in the afternoon before testing. This suggests that there must be another component of the circadian rhythm, except sleep, that affects cardiovascular responses to exercise. PD patients tend to be of advanced age and are less able to engage in an appropriate amount of cardiovascular training; the subsequent deconditioning might also potentiate OH (Jost, 2012). No studies have assessed exercise responses in PD in the home setting over 24 h. In order to confirm exercise-induced hypotension or post-exercise OH, an incremental exercise test preceded and followed by a HUT and/or standing test is the gold standard.

### Drug induced hypotension

There are several reasons why OH may occur in PD, but one crucial factor might be PD medication due to its cardiovascular side effects. Levodopa is known to have a lowering effect on BP and may cause or exacerbate OH (Hoehn, 1975; Jamnadas-Khoda et al., 2009) because it reduces stroke volume, cardiac output, and systemic vascular resistance (Jamnadas-Khoda et al., 2009). Any symptoms relating to PD medication can be detected by conducting a 24 h ABPM when keeping a detailed autonomic protocol/diary and noting the time of day when medication was taken.

### PD and BP “dipping”

There are few published studies on the presence and extent of non-dipping in PD. There often is a correlation between the severity of OH and non-dipping in PD (Senard et al., 1992). A recent study found that 95% of PD patients who were diagnosed with OH were non-dippers, compared to 79% (of 40) of PD patients who did not have OH (Sommer et al., 2011). Furthermore, it has been found that patients with OH tend to have a higher BP variability then those without OH (Senard et al., 1992).

Another factor potentially influencing the circadian BP rhythm is medication. PD patients, who are also diagnosed with OH and therefore treated with fludrocortisone, tend to have higher BP readings and are therefore more prone to being non-dippers than those PD patients who do not take fludrocortisone (Hakamaki et al., 1998).

**Chart 1:**
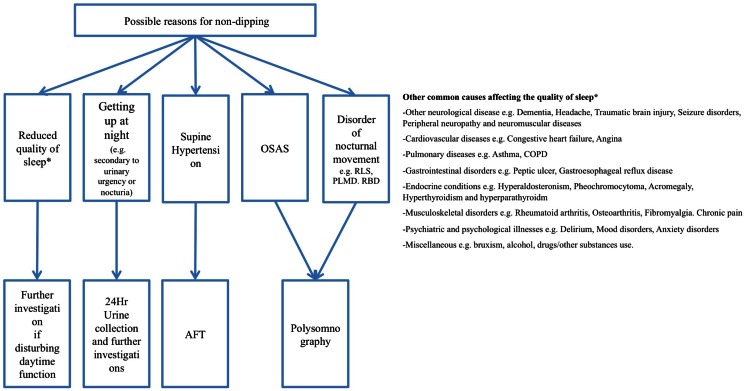
**Overview of possible reasons for non-dipping and how to diagnose them**.

Ambulatory blood pressure monitoring findings in PD patients are in stark contrast to ABPM measurements in patients with essential hypertension, who only show a reversed circadian rhythm in 15% of all cases compared to 48–93% (Ejaz et al., 2006; Schmidt et al., 2009) in PD patients. Nonetheless, there seems to be a correlation between hypertension and non-dipping in PD, since Oh et al. (2012) found that in PD patients the percentage of those with hypertension was higher in the non-dipper group than in the dipper group. Non-dipping in PD can be caused by a variety of factors, which are discussed in this review and summarized in Chart 1.

**Chart 2:**
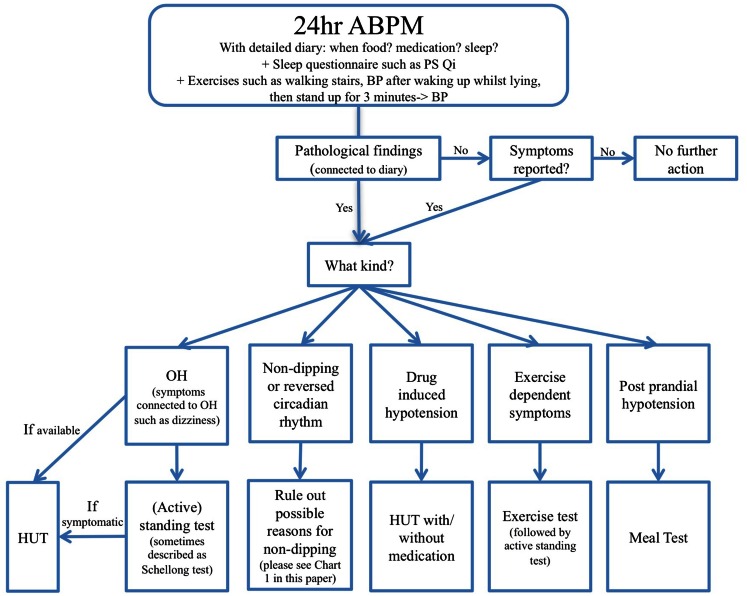
**Twenty-four hour ABPM monitoring flow chart for PD**.

### Supine hypertension

Supine hypertension, which is defined as a systolic BP ≥150 mmHg or diastolic BP ≥90 mmHg (Goldstein et al., 2003b) is quite a common feature of autonomic failure syndromes (Goldstein et al., 2003b). This can occur at any time during the day when the patient is lying down or even semi-recumbent, but is especially prevalent at night during sleep. These patients will appear as non-dippers when administering a 24-h ABPM.

It has been suggested that the main reason for supine hypertension is increased vascular tone in patients with autonomic failure (Jordan and Biaggioni, 2002). But this can vary in different conditions. In MSA, for example, supine hypertension is proposed to be caused by residual sympathetic tone unrestrained by the lack of baroreflex buffering, whilst in PAF the causes of hypertension are not known but are less dependent on sympathetic tone (Shannon et al., 2000). Supine hypertension is often linked to OH, and in one study looking at 24 patients who had PD with OH, all patients had SH, whilst 27 patients with PD without OH showed a normal BP profile (Goldstein et al., 2003b). An explanation for these findings could be that as well as anti-OH therapy with fludrocortisone, PD treatment with could possibly cause supine hypertension as a side effect (Goldstein et al., 2003b). But generally it is probably due to prolonged disease duration and decreased cardiovascular autonomic function.

It is of high importance to recognize supine hypertension in PD patients, since nocturnal hypertension is linked to an increased cardiovascular risk (Sharabi and Goldstein, 2011). As yet, there has been no study looking at supine hypertension in PD in a home setting using 24 h ABPM.

### PD and sleep

Several factors might lead to a higher BP at night in PD, which might include problems relating to sleep. A bad quality of sleep might cause non-dipping. Likewise, it could also be caused by nocturia (getting up and walking to the bathroom at night) or nocturnal movement disorders (see below).

It is well known, that up to 90% of PD patients suffer from sleep disruption (Chaudhuri et al., 2006; Videnovic and Golombek, 2012), this was described as early as in the “Essay on the shaking palsy” (Parkinson, 1817). Sleep dysfunction predominantly occurs in the early stages of the disease and may even predate it by several years (Chaudhuri et al., 2006; Videnovic and Golombek, 2012). A reason for a high incidence of sleep disorders in PD may be that dopamine, which is depleted in PD patients, plays a crucial role in sleep regulation and circadian homeostasis (Videnovic and Golombek, 2012). Dopamine depletion may furthermore lead to abnormal sleep-wake cycles which may cause extreme daytime sleepiness (Rye, 2004). It is essential to ask PD patients detailed questions about their quality of sleep when conducting a 24-h ABPM, to find possible reasons for non-dipping, but also to evaluate quality of sleep. An in-depth sleep questionnaire, such as the Pittsburgh sleep quality index (PSQI) (Buysse et al., 1989), which has been used successfully in large scale PD studies (Louter et al., 2012), could therefore be used to objectively assess quality of sleep in PD patients that undergo 24 h ABPM.

While in-depth discussion of sleep disorders in PD is beyond the scope of this review, it should not be neglected. Readers are directed to other excellent reviews (Garcia-Borreguero et al., 2003; Comella, 2007; Peeraully et al., 2012).

### Disorders of nocturnal movement in PD

Additional findings that may be made through an ABPM are disorders of nocturnal movement. These would be reflected in nocturnal non-dipping, since they often keep patients awake or do not allow for deep sleep and relaxation. Excessive nocturnal movement is fairly common in PD (Rye, 2004). These movements are involuntary movements, which are associated with the extrapyramidal motor system (Rye, 2004). But there may be tremor, aperiodic and periodic limb movements, as well as increased phasic and tonic electromyographic activity in REM sleep, which can often be found in neurodegenerative diseases involving nuclei of the basal ganglia (Rye, 2004). It has been reported that roughly a fifth (20.8%) of PD patients suffer from restless leg syndrome at night which is a comparably high number in contrast to a prevalence of 8–10% in the aged-matched general population (Ondo et al., 2002). In addition, REM sleep behavior disorders have been found to occur in 15–30% of PD patients when evaluating bed partner accounts of PD patients (Comella et al., 1998).

### PD and obstructive sleep apnea syndrome

Some papers suggest a connection between PD and obstructive sleep apnea syndrome (OSAS), with an OSAS prevalence of up to 43% in PD patients (Diederich et al., 2005). This may be related to impairment of breathing control, impaired respiratory muscle function due to rigidity and faulty autonomic control of the lungs, fluctuating muscle functioning, laryngeal spasm associated with off-states or upper airway dysfunction with tremor-like oscillations (Hening et al., 2009). However, a more recent study found, in contrast to earlier investigations, that sleep apnea was less frequent in PD then in normal controls, suggesting that OSAS may not be of major clinical relevance in PD patients (Cochen De Cock et al., 2010). Regardless of the contradicting studies on prevalence in PD, OSAS might severely affect quality of sleep as well as nocturnal BP profiles of PD patients (Apps et al., 1985; Trotti, 2010), and therefore confound ABPM findings by producing a non-dipper profile, through sleep disruption and elevations in BP (Silverberg et al., 2002), which constitute a cardiovascular risk factor.

### Management of supine hypertension in PD with OH

When a defective circadian rhythm has been detected, therapeutic strategies can be developed, which can include both non-pharmacological and pharmacological measures. The most obvious management of high BP at night would be anti-hypertensive medication but this is not advisable for patients with autonomic dysfunction or autonomic failure who typically experience or are at high risk of OH. The EFNS guidelines for the treatment of supine hypertension recommend conservative measures as used in the therapy of OH, including raising the head end of the bed by 20–30 cm, or 10°, or refraining from taking pressor medication, such as midodrine, after 6 p.m. (Lahrmann et al., 2006), and to take fludrocortisone only in the morning and at lunch time. Occasionally, short acting anti-hypertensive medication such as sublingual nitro-glycerin might be a good choice (Lahrmann et al., 2006). Other lifestyle changes such as using the effects of the postprandial BP drop may be beneficial as well. Consensus guidelines of when and how to treat supine hypertension in PD do not yet exist.

The treatment of OH in PD is beyond the scope of this review. In summary, OH symptoms in PD can in many cases be ameliorated by non-pharmacological measures that aim to expand blood volume, increase venous return, and/or reduce/avoid known risk factors for OH. Usually, a multifaceted approach combining non-pharmacological and pharmacological treatment is needed. For further information the reader is directed to other excellent and extensive publications (Lahrmann et al., 2006; Bannister and Mathias, 2013).

## Summary

Abnormalities in cardiovascular autonomic function such as OH or supine hypertension, are common complications in PD and should therefore be diagnosed and treated correctly. Performing 24 h ABPM in a home setting offers an affordable, widely available, and valuable procedure to gain an overview of cardiovascular autonomic dysfunction and its potential causes. It especially gives insight into BP variations throughout the day, which may be affected by various factors such as posture, food, exercise, and medications. Even when a HUT is negative, a 24-h ABPM may provide important information about how BP changes during a patient’s routine day and can even unmask cardiovascular autonomic dysfunction. Furthermore, it can also provide crucial information about the circadian rhythm of BP and HR and possible sleep disruption, which are common symptoms in PD. The results of a 24-h ABPM rely on the precise implementation of the method and it is recommended that the patient completes a detailed autonomic protocol/diary of their activities in order to be able to link results, symptoms, and events that may occur during the day. Research on the use of 24 h ABPM in PD so far is limited but initial findings suggest that an abnormal circadian BP rhythm in PD is common with an incidence in the range of 40–93%; the mechanisms of which may be due to autonomic dysfunction/failure, disrupted sleep, supine hypertension, OSAS, or disorders of nocturnal movement.

In order to summarize what has been described above, the following flowchart provides an overview of possible findings in a 24-h ABPM in PD patients. It furthermore illustrates which diagnostic conclusions could be drawn from the findings. This flow chart cannot cover all possible findings and diagnoses in a 24-h ABPM, but summarizes what has been described in this review. It demonstrates the vast possible findings of 24 h ABPM which highlights the usefulness of administering 24 h ABPM as a regular screening tool in PD patients to minimize and treat symptoms of autonomic nervous system dysfunction.

## Conflict of Interest Statement

The authors declare that the research was conducted in the absence of any commercial or financial relationships that could be construed as a potential conflict of interest.

## Abbreviations

24h-BP, 24 hour blood pressure measurement; ABPM, ambulatory blood pressure measurement; AFT, autonomic function test; BP, blood pressure; HDRS, Hamilton Rating Scale for Depression; HR, heart rate; HUT, head-up-tilt; l-DOPA, levodopa; l-OH, late orthostatic hypotension; MAP, mean arterial pressure; MIBG, (123) I-metaiodobenzylguanidine; MSA, multiple system atrophy; OH, orthostatic hypotension; OSAS, obstructive sleep apnea syndrome; PAF, pure autonomic failure; PD, Parkinson’s disease; PPH, postprandial hypotension; PVN, paraventricular nucleus; SCN, suprachiasmatic nuclei.
